# Thermosensitive In Situ Gels for Joint Disorders: Pharmaceutical Considerations in Intra-Articular Delivery

**DOI:** 10.3390/gels8110723

**Published:** 2022-11-08

**Authors:** Marina Koland, Anoop Narayanan Vadakkepushpakath, Anish John, Arunraj Tharamelveliyil Rajendran, Indu Raghunath

**Affiliations:** Department of Pharmaceutics, NGSM Institute of Pharmaceutical Sciences (NGSMIPS), Nitte (Deemed to be University), Mangalore 575018, India

**Keywords:** thermosensitive, intra-articular, gels, poloxamer, joint disease

## Abstract

The intra-articular administration of conventional drug solutions or dispersions in joint diseases such as osteoarthritis has a relatively short retention time and, therefore, limited therapeutic effect. Thermosensitive polymer solutions that exhibit a sol–gel phase transition near body temperature after injection can prolong drug retention by providing a depot from which the drug release is sustained while relieving inflammation and preventing degradation of the joint complex. Thermosensitive hydrogels have in recent times garnered considerable attention in the intra-articular therapeutics of joint diseases such as osteoarthritis. Among the stimuli-responsive gelling systems, most research has focused on thermosensitive hydrogels. These gels are preferred over other stimuli-sensitive hydrogels since they have well-controlled in situ gelling properties and are also easier to load with drugs. Temperature-sensitive polymers, such as block copolymers or poloxamers, are frequently used to modify their gelation properties, usually in combination with other polymers. They are compatible with most drugs but may pose formulation challenges in terms of their low-response time, highly fragile nature, and low biocompatibility. The stability and biodegradability of implant hydrogels can control the drug release rate and treatment efficacy. This review stresses the application of thermosensitive gels in joint disorders and summarizes recent developments for intra-articular application, including the incorporation of nanoparticles. The hydrogel composition, drug release mechanisms, and the challenges involved in their formulation and storage are also discussed.

## 1. Introduction

Several acute and chronic inflammatory diseases of the joints affect individuals. The most prevalent joint disorders are osteoarthritis, rheumatoid arthritis, and gouty arthritis [1]. Severe chronic diseases such as rheumatoid arthritis are debilitating and even destructive enough to the joints to require surgery for replacement [2,3]. The commonly used drugs for the treatment of joint disorders include nonsteroidal anti-inflammatory drugs (NSAIDs), COX-2 enzyme inhibitors, corticosteroids, disease-modifying osteoarthritis drugs (DMOADs), and disease-modifying anti-rheumatic drugs (DMARDs) such as methotrexate, which are systemically administered. Some of these drugs are also occasionally injected into the joints (intra-articular injection) to treat synovitis, inflammation, and pain.

Intra-articular (IA) drug delivery is often preferred when the joint disease is severe and painful since it places high concentrations of the drug directly at the desired site, with a faster onset of action and increasing its bioavailability while reducing side effects associated with systemic delivery [4]. For pain management and joint lubrication, the most common drugs administered by IA are corticosteroids and hyaluronic acid (HA) [5]. However, intra-articular drug delivery for treating joint diseases has always been challenging. The retention of the drugs at the site of injection is poor, and they are rapidly cleared from the site. Small drug molecules are removed from the joint tissue by rapid uptake into the synovial capillaries and lymphatics [6].

Researchers have reported different strategies for increasing the local retention of the intra-articularly administered medication. They have designed drug delivery systems with slow-release effects that prolong the duration of action or reduce clearance from the administration site. These formulations include microspheres [7], nanoparticles [8], liposomes [9], and gels [10].

The IA administration of drug-incorporated hydrogels has drawn much attention recently. Their viscosity allows retention of the drug at the joint site, enables sustained release of the drug into the surrounding tissues, and provides prolonged effects. Their higher water content has a lubricating and soothing effect on the inflamed tissues, mimicking the soft tissues within the synovium [11].

**Figure 1:**
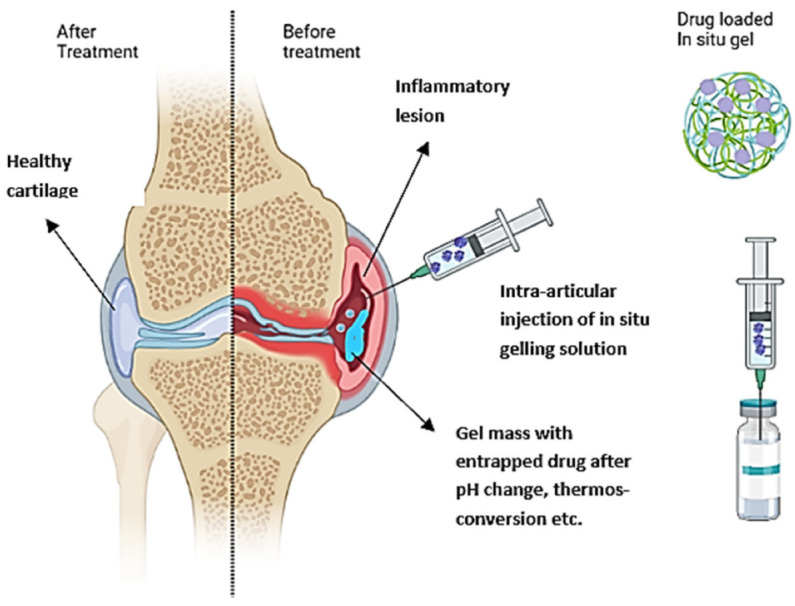
Schematic representation of the IA administration of an in situ gelling drug formulation into inflamed osteoarthritis (OA) joint and healthy tissue after treatment.

Of particular interest in IA delivery of gels are in situ forming hydrogels, which can be injected as solutions and suspensions but gel under physiological conditions such as a change in pH, temperature, or ionic composition (Figure 1). The viscoelastic properties of the system enable the measurement of precise doses before administration and retention in the joints after injection due to gelation [12]. The gelation at the injection site provides a depot for the slow and continuous release of the incorporated drug. Such formulations increase drug bioavailability and achieve high local drug concentrations while minimizing systemic toxicity by decreasing dosing frequency.

Thermosensitive or thermoreversible in situ hydrogel systems are sols below body temperature and turn to gels after administration into the body. The principal polymers used to prepare such systems are Poloxamers or Pluronics, e.g., Poloxamer 407 (Pluronic F127) and Poloxamer 188 (Pluronic F68). Poloxamers are biocompatible, nonionic triblock copolymers made of complex hydrophilic and lipophilic blocks. A typical triblock copolymer has two blocks of hydrophilic poly (ethylene oxide) (PEO) and a central hydrophobic poly (propylene oxide) (PPO) block, i.e., polyoxyethylene-polyoxypropylene-polyoxyethylene (PEO-PPO-PEO) [13]. Thermoreversible gels are formed from concentrated aqueous solutions of these poloxamers due to the dehydration of polymer blocks with increased temperature [14]. A rigid and highly viscous hydrogel is formed due to such nonchemical crosslinking.

Poloxamer-based thermoreversible hydrogels have the necessary physicochemical properties to successfully administer therapeutics in joint disorders. Besides their nontoxic and biodegradable nature, poloxamers can also enhance the aqueous solubility of poorly soluble drugs [15]. The low viscosity of the solutions allows for the ease of injection.

This review presents an overview of developments in thermosensitive gelling systems for the IA administration in joint diseases. We have started with a brief discussion of various kinds of intra-articular actives which could be administered as thermosensitive hydrogels. Attempts have been made to focus on the pharmaceutical considerations in the formulation of such systems such as their compositions and choice of polymers and mechanisms of gelation and drug release. We have also discussed the inclusion of various types of nanocarriers and their biomedical applications, specifically in joint disease therapeutics. Finally, this paper also highlights the challenges involved in formulating, evaluating, and stabilizing thermosensitive gels.

## 2. Intra-Articular Administration of Therapeutics

Intra-articular therapy includes long-term medications such as NSAIDs, corticosteroids, and other biologics [16]. In situ forming hydrogels are a simple and effective method to prolong the retention of drugs in the joints and their duration of action. Several drug delivery technologies are often combined with in situ gelling systems. Biodegradable nano-drug delivery systems are nontoxic, enhancing drug stability and delivering the drug in a controlled manner [17]. Utilizing micro- and nanoparticles increases the medication’s retention time at the joint, maintains the drug concentration at the desired level through a controlled release mechanism, and eliminates the potential side effects of the drug through low-dose administration. On the other hand, micro-sized particles are easily ingested by macrophages in synovial linings, but nano-sized particles readily escape from the joint cavity [18]. In situ forming hydrogels for intra-articular administration have only recently been launched. Many studies point to the possibility of hydrogels being used to treat osteoarthritis. After that, some treatment strategies and hydrogel formulation designs containing NSAIDs/bioactive molecules suitable for IA therapy are discussed.

### 2.1. Types of Intra-Articular Treatments in Joint Diseases such as Osteoarthritis

Intra-articular therapy (IAT) is a restorative procedure that healthcare experts widely utilize. IAT helps individuals with joint synovitis, effusion, and discomfort from inflammatory arthritis and osteoarthritis [19]. Treatments for osteoarthritis (OA) can be divided into surgical, pharmacological, and nonpharmacological [20]. Patient education and self-management are two nonpharmacological modalities recommended by the European League Against Rheumatism (EULAR). Other nonpharmacological modalities include naturopathy, acupressure, walking aids, shoe and insole modification, and electromagnetic therapy [21]. These are beneficial; nevertheless, many patients fail to complete the treatment over time. Marrow stimulation, arthroscopic debridement, and articular cartilage replacement are utilized to heal cartilage defects and degeneration. These procedures are very sophisticated and depend on healthy cartilage [22]. Pharmacological therapies include symptomatic slow-acting drugs (glucosamine and chondroitin sulfate), opioid analgesics, nonsteroidal anti-inflammatory drugs (NSAIDs), and IA injections of substances such as corticosteroids, blood-derived products, and HA [23]. Osteoarthritis Research Society International (OARSI) recommends considering side effects while choosing medications for osteoarthritis patients. New treatments, such as nerve growth factor (NGF) antibodies, have been evaluated and have shown positive results in reducing pain in patients with hip and knee OA [24]. Patients in the late stages of OA, especially young, active patients with intermediate radiographic severity in OA, may benefit from surgical treatments, including joint replacement surgery, knee osteotomy, and knee-joint separation [25]. To give this, clinicians must have complete knowledge of all appropriate treatment options, including their risks, availability, and cost effectiveness [26].

#### 2.1.1. Nonsteroidal Anti-Inflammatory Drugs (NSAIDs)

NSAIDs are the pharmacological treatment of choice for osteoarthritis (OA). Numerous placebo-controlled trials have demonstrated that NSAIDs provide higher pain relief than placebo, with standardized mean differences in pain and function ratings of 0.33 standard deviations, indicating a moderate benefit [27]. Topical NSAIDs, for example, were strongly suggested for people with knee OA (Level 1A: 75% in favor and >50% strong recommendation). COX-2 inhibitors were indicated at Level 1B for people with gastrointestinal comorbidities, and NSAIDs were recommended at the second level; oral NSAIDs were not advised for those with cardiovascular comorbidities or infirmity. The use of corticosteroids has the drawback of inducing cartilage degeneration over time; as a result, NSAIDs could be an alternative to administering corticosteroids [28]. Berrin et al. developed an in situ gelling hydrogel containing nanoparticles for the prolonged local delivery of diclofenac sodium via emulsion–solvent evaporation. Compared to the conventional delivery system, the system described above released the drug at a controlled rate over thirty days. This slow release was accomplished by combining the formulations of polymeric nanoparticles and poloxomer 407-chitosan in situ hydrogels [29].

#### 2.1.2. Hyaluronic Acid

Nonsulfated hyaluronic acid (HA) is a naturally occurring glycosaminoglycan with distinct physicochemical properties; it is made up of D-glucuronic acid and N-acetylglucosamine units that are repeated in an alternating fashion. HA is found in rooster combs, shark skin, bovine eyeballs, bovine nasal cartilage, rabbit brain, rabbit heart, etc. [30]. Viscous supplementation using intra-articular (IA) injections of HA has been widely and successfully utilized from 1987 to 1988 in Japan and Italy [31]. The intra-articular HA treatment typically entails a series of injections, administered by a specialized physician, and spaced apart by one week. Since 2004, a single-injection IA HA therapy (3 mL, 60 mg) has been suggested as an alternative to the multi-injection regimen (currently 3 × 2 mL, 20 mg per injection), dispensing the same quantity of HA. A single injection would reduce the number of medical visits, intrusive procedures, and other associated risks [32]. HA increases the viscosity and elasticity of synovial fluid. Synovial fluid with an average HA concentration works as a viscous lubricant and an elastic shock absorber during slow joint movements. Studies showed that treatment of HA produces chondroprotection, which is caused by HA binding to CD44, which inhibits interleukin (IL)-1β expression, leading to a decline in matrix metalloproteinase (MMP) −1, 2, 3, 9, and 13 production [33,34]. Liling et al. recently developed in situ gelling nanosystems for combined therapeutic efficacy. By co-loading hyaluronic acid (HA) and celecoxib, formulated as the HLC precursor, this study created a lyotropic liquid crystal (LLC) precursor. Medication retention in the articular cavity is effectively prolonged, leading to an anti-inflammatory effect that lasts longer. According to rheological tests, HLC gel with a cubic lattice structure has a spring-like effect to cushion joint shock. It is promising to protect cartilage by preventing mechanical wear and tear, lubricating joints, and reducing intense stress (about 50 percent). In vivo degradation studies proved that the system was biocompatible and biodegradable [35]. To summarize, intra-articular HA injections are acceptable, may be effective, and may alleviate pain in mild OA of the knee for up to 170 days.

#### 2.1.3. Corticosteroid Injections

White and Norton launched the first clinical testing of IA corticosteroid injection in 1958, which the American College of Rheumatology currently supports [36]. Corticosteroids possess immunosuppressive and anti-inflammatory properties, but their pharmacological action is complicated. The Food and Drug Administration (FDA) has approved five injectable corticosteroids for IA injections. Methylprednisolone acetate, triamcinolone acetate, triamcinolone hexacetonide, betamethasone acetate, betamethasone sodium phosphate, and dexamethasone are among them. Through their direct action on nuclear steroid receptors, corticosteroids suppress the inflammatory and immunological response at several points [37]. Multiple studies have found that intra-articular corticosteroid injections have a duration of effect that can last anywhere from 1 week to 24 weeks. There is unanimity that steroid injections provide around one week of relief to patients. Corticosteroid injections might have harmful side effects. Joint infection after using corticosteroids is a very uncommon occurrence. Skin atrophy, pain, tendinopathy, and systemic hyperglycemia are just some of the additional issues that may arise and the mortality rate is estimated to be around 11% [38].

#### 2.1.4. Platelet-Rich Plasma (PRP)

Since the 1990s and more recently, platelet-rich plasma (PRP), a naturally occurring mixture of highly concentrated platelets, associated growth factors, and other bioactive components produced by centrifugal separation of whole blood, has been used in maxillofacial and plastic surgery to treat bone, tendon, and ligament injuries [39]. The various solutions commonly contain PDGF, IGF-1, and TGF-B as their growth factors. Other growth factors may also be present. Studies using PRP have shown some degree of anabolic effects on chondrocytes, such as the deposition of type II collagen. IGF-1 promotes the synthesis of type II collagen, proteoglycans, and other extracellular matrix components in the joint, contributing to its anabolic effects. These components can enhance adhesion between chondrocytes and prevent proteolysis in the milieu of the extracellular matrix. IGF-1 is responsible for stimulating the production of type II collagen, proteoglycans, and other extracellular matrix components in the joint. On chondrocytes, PDGF and TGF-beta both have anabolic effects. However, the rise in chondrocyte production is brought on by PDGF. Pro-inflammatory cytokines such as NF-kB and IL-1 have dropped in PRP. TGF-beta also promotes the differentiation of stromal cells and mesenchymal stem cells into chondrocytes [40]. The most prevalent adverse effects were pain at the injection site, joint stiffness, vertigo, headache, nausea, dyspepsia, perspiration, and tachycardia [41]. With PRP, pain relief seems to start about two months after injection and could last for up to a year. Compared to hyaluronic acid, PRP was found to be beneficial based on ratings from the international functional knee documentation committee (IKDC) and VAS pain scores; however, publication bias may exist. Additionally, these advantages might be enhanced in younger patients and those who have diseases that specifically affect cartilage tissue [37].

#### 2.1.5. Stem Cell Therapy

Mesenchymal stem cells (MSCs) are a possible source for the therapy of OA due to their ability to differentiate into chondrocytes and regulate the immune system [42]. MSCs were first isolated from bone marrow and later from other tissues, such as the placenta, umbilical cord, cord blood, amniotic fluid, dental pulp, and adipose tissue [43]. There are three essential elements for cartilage tissue engineering: cells, scaffold, and environment. Adult stem cells, specifically multipotent mesenchymal stem cells, are the cell type of choice for tissue engineering due to their ease of isolation, expansion, and multilineage differentiation potential. MSCs can be employed as progenitor cells to create cartilage implants that can heal chondral and osteochondral lesions and as trophic generators of bioactive molecules to stimulate endogenous regeneration processes in the OA joint [30]. MSCs have been successfully employed in preclinical models to resurface degenerated cartilage. In early phase clinical trials, intra-articular (IA) administration of MSCs leads to pain reduction and cartilage protection or healing [44]. Direct IA injection of MSCs would eliminate surgeries and adverse effects such as periosteal hypertrophy and ossification, immunological reactivity, and disease transmission from xenograft coverage. More relevantly, the injection is simple and easy to use, which may lead to better treatment options, especially for older people with multiple health problems. Despite this promise, no clinical studies have been carried out; only a few case reports have been published. In situ hydrogel-forming materials provide an extracellular matrix for administered stem cells. The matrix offers a favorable environment for the cells with mechanical support [45].

Various therapeutics administered by IA injection in the management of joint diseases are summarized in Table 1.

## 3. Thermosensitive Gels and Their Comparison to Other Stimuli-Sensitive Gels

Endogenous and exogenous triggers activate stimuli-sensitive gels. The change in pH, temperature variation, ultraviolet radiation, solvent exchange, and the presence of ions or molecules are a few of the stimuli that can cause the sol–gel transition [48]. Endogenous triggers such as pH, enzyme activities, temperature, and redox levels can also modulate the release of the drug from the smart gels. External stimuli such as light, magnetic energy, and ultrasound can also control the drug release at the application site. Stimuli-sensitive nanovesicles can modify the kinetics of encapsulated drugs by controlling the burst or unexpected release behavior [49].

Thermoresponsive in situ hydrogels are prepared from polymers whose physical properties change with increasing temperatures. With the right combination of thermosensitive polymers, the resulting composition can exhibit gel transition at body temperature (37 °C) [50]. This is achieved with the help of triblock polymers which contain both hydrophilic and hydrophobic chains in the structure. Thermosensitive hydrogels are reliable systems for formulating hydrophilic and hydrophobic drugs because of their biocompatibility and biodegradability. Studies show that thermosensitive gels can control drug release over several days and are suitable for delivery via mucosal, transdermal, ophthalmic, oral, buccal, intranasal, intravesical, intravaginal, transdermal, and local injection [51]. Because thermosensitive gels can replicate the three-dimensional microenvironment of cells, they can be used to repair tissues, including cartilage and wounds [52]. The mechanical strength and stability of thermosensitive gels can be improved by UV irradiation [53].

For IA applications, the gelling materials used for the formulation themselves have a protective effect on the cartilage [50]. A thermosensitive hydrogel based on methacrylate chondroitin sulfate and a glycol triblock copolymer has been shown to be a promising biomaterial for cartilage 3D printing [54].

pH-responsive systems target the drug into the low pH regions of bone due to the high amount of lactate production in the inflamed joints. This acidic environment acts as a guiding mechanism for the delivery system to release the drug in osteoarthritic conditions [55]. Few studies reported that significant swelling and drug release were found at low pH (7.4) [56]. Hence, the system may not work properly due to the wrong or improperly activated signaling systems. Sometimes the poor biocompatibility and less degradation of the polymeric materials make the systems unsuitable to work with pH responses in the inflamed joints [57].

Magnetic responsive gels have magnetic components embedded in a hydrogel matrix. The magnetic field can externally control the physical, chemical, and mechanical properties of the gel. The characteristics of the magnetically controlled hydrogel are determined by the magnetic content, concentration, size, and shape of the magnetic particle [58]. The drug release of iron oxide-based gels is controlled remotely by a magnetic field. Although there are a few advantages to this drug delivery system, the two main limitations are the long response time and less precisely controlled architectures of these stimuli-responsive smart biomaterials [59].

An ultrasound-activated hydrogel system has been reported for cancer treatment as well as tissue engineering. Micelles, nanobubbles, nanodroplets, emulsions, and vesicles are created with acoustic-energy-controlled drug delivery, which can work either thermally or nonthermally. When exposed to an ultrasound trigger, ultrasound-responsive hydrogels release the drug to the target. To avoid damaging the surrounding tissues of the drug release area, the amount of ultrasound required to trigger the release should be kept to a minimum [60].

Bone disorders can be treated with drug delivery methods that respond to enzymes. In order to promote the formation or degradation of the hydrogel network, enzyme-responsive hydrogels typically contain enzyme-responsive polypeptides. Hydrogels responsive to various enzymes such as phosphatase, metalloproteinase, and tyrosinase have been reported [61]. One of the inflammatory-related enzymes that are primarily secreted in OA is MMP. Triglycerol monostearate (TGMS) polymers were investigated by He et al. for their potential to deliver medications in response to enzymes in OA mice [62]. One of the limitations of enzyme-responsive gel drug delivery is that due to the nonenzymatic hydrolysis of polymers (e.g., TG-18) in aqueous environments, the drug release wavers [63].

Compared to most kinds of stimuli-responsive hydrogels, thermoreversible gels lend themselves to simple modifications in composition to control gel formation at physiological temperatures. Hydrophilic and hydrophobic drugs have been loaded into these gels without compatibility problems [51]. Unlike pH-responsive gels, the stability of thermosensitive systems is less likely to be affected by changes in pH.

Different stimuli-responsive hydrogels with their advantages and disadvantages are listed in Table 2.

Gelling materials are compatible with various hydrophilic and hydrophobic drugs and excipients like acids (hyaluronic acid), metals (Copper) and blood components (platelet-rich plasma-PRP). Hence gels are formulated to target various pharmacological responses. Because of the compatibility with different types of polymers and drugs, the formulation of multifunctional gels for the treatment of OA is possible [64].

However, the practical translation of these environmental-sensitive hydrogels is still facing challenges in terms of low-response time, highly fragile nature, and low biocompatibility.

Multiple mechanism functioning is one of the methods used to overcome the challenges faced during the formulation of effective drug delivery from smart hydrogels. The combination of exogenous and endogenous triggers has the potential to overcome the limitations of stimuli-responsive hydrogel drug delivery. Hydrogels that are activated by both temperature and pH responses are a good example [65].

## 4. Temperature-Sensitive Materials and Gelation Mechanism

Thermosensitive hydrogels are more suitable for applications involving drug delivery. Several hydrophilic polymers combine to form insoluble hydrophilic three-dimensional networks that appear to swell in aqueous media, forming solid-like structures with fluidity at the microscale. The delicate balance between the hydrophobic and hydrophilic parts of the monomer results in the phenomenon of thermal response. Typically, thermosensitive polymers contain both hydrophobic and hydrophilic components in their structures. Temperature alters how hydrophilic and hydrophobic segments interact with water molecules, changing the crosslinked network’s solubility and the sol-gel phase transition. The gelation process is reversible with changes in the surrounding temperature [66].

**Figure 2:**
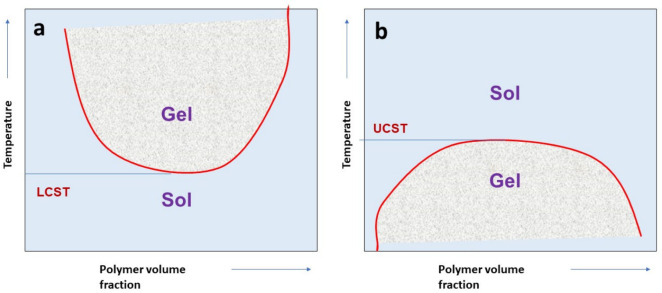
Sol-gel phase transition behavior of thermosensitive polymers (**a**) at lower critical solution temperature (LCST) and (**b**) at upper critical solution temperature (UCST).

Thermosensitive hydrogels are divided into positive and negative thermosensitive hydrogels based on their gelation behavior. The sol-gel transition behavior of these polymers is graphically represented in Figure 2.

### 4.1. Positive Thermosensitive Hydrogels

Hydrogels with positive thermosensitivity have an upper critical solution temperature (UCST). Below the UCST, the thermosensitive hydrogels could contract and remain in the solution. Changes in the system’s enthalpy drive this gel-to-sol transition. As the ambient temperature decreases, a variety of natural polymers, such as agarose, gelatin, and amylose, may undergo temperature-dependent sol-to-gel transition. Consequently, they have limited utility in hydrogel-based drug delivery.

### 4.2. Negative Thermosensitive Hydrogels

Negative thermosensitive hydrogels possess lower critical solution temperatures in contrast to positive thermosensitive hydrogels. Due to their gelation behavior above the lower critical solution temperature (LCST), negative thermosensitive hydrogels are commonly used to formulate in situ gelling systems for sustained drug release. Negative thermosensitive hydrogels remain in sol form below LCST and transform gradually into a gel above LCST because of increased entropy within the system. The higher entropy of water molecules when the polymer is in a swollen state produces a hydrophobic effect, resulting in gelation [66,67].

In situ gelling intra-articular drug delivery systems favorably use this sol-gel transition above the LCST. The most popular of these is poly (N-isopropyl acrylamide), or PNIPAAm, which has an LCST close to 32 °C and is useful for intra-articular applications because it stays as a homogeneous solution in storage conditions and gels at body temperature (37 °C) after injection [67]. The volume phase transition temperature (VPTT) and behavior of thermosensitive hydrogels/microgels can typically be modified by adjusting the ratio of hydrophilic to hydrophobic groups or by imbuing the polymer with an electrostatic charge that would affect the interactions between polymers and water [67,68]. Several monomers are used in the synthesis of PNIPAAm copolymers with modified thermoresponsive behaviors. These monomers include various cationic, anionic, and neutral molecules such as N-vinyl formamide, 4-vinyl pyridine, 2-(aminoethyl)-methacrylate hydrochloride, N-N-(diethylamino) ethyl methacrylate, acrylic acid, methacrylic acid, vinylacetic acid, allylacetic acid, acrylamide, etc. [68].

**Figure 3:**
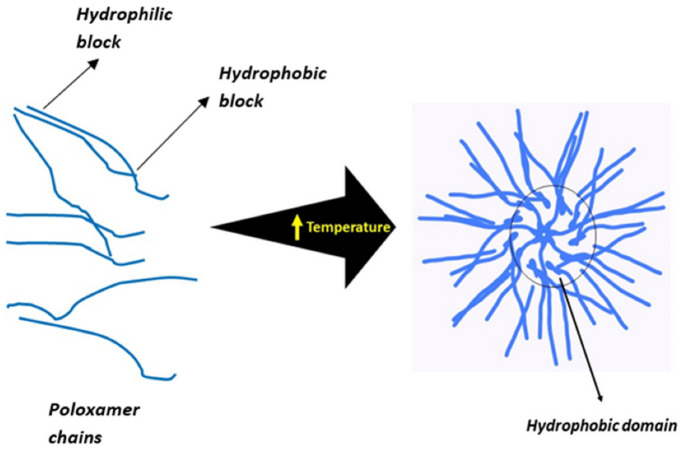
Mechanism of a temperature-sensitive system [69].

Poloxamers (Poly (ethylene oxide)98–poly (propylene oxide)67–poly (ethylene oxide) 98) are (PEO–PPO–PEO) triblock copolymers used extensively for their thermoresponsive behavior. It has been reported that poloxamer can form a gel in aqueous solutions by micelle formation based on its critical micelle concentration and temperature (Figure 3). PEO/PPO micelles form as the temperature rises. A hydrophobic core is created by wrapping the PPO chain inside. The hydrophobic PPO is encased in a layer of hydrophilic PEO that interacts with water molecules. Additionally, the PEO/PPO micelles will communicate with one another. If the concentration of the poloxamer solution is higher than its critical micellar concentration, strong interactions will cause the micelles to entangle, pack, and gather, turning them into a polyethylene glycol (PEG) poly (lactic acid) co-(glycolic acid) (PLGA) block, with copolymers that are also thermosensitive [66].

A thermosensitive gel based on poloxamer increases the gelling viscosity and gel dissolution time with sustained drug release [70,71]. Tanmoy Das et al. developed a novel, biodegradable, injectable, in situ gel system of methotrexate by varying Pluronic F-127 and xanthum gum concentrations. The gel showed thermosensitivity and was thermoresponsive depending on the concentration and composition of polymers. An improvement in an inflammatory condition and a reduction in dose and dosing frequency was achieved in managing rheumatoid arthritis [72]. A novel, biodegradable injectable in situ gel of methotrexate was developed by varying concentrations of Pluronic F 127 (20 and 22%) and xanthan gum (0.2 to 0.6% w/v). The polymer concentration and composition influenced the drug release from the gel with thermosensitivity [73]. Thermosensitive hydrogels of etanercept were prepared with chitosan and Pluronic F-127 and β-glycerolphosphate. The hydrogel had a protective effect on cartilage with a modified drug release [50].

Intra-articular delivery of methotrexate using PEP thermosensitive hydrogel decreased the clearance rate from the joint cavity and prolonged the drug release. The study was conducted by administering the formulation in rats. Methotrexate concentration in plasma served as an indicator for drug release from the intra-articular injected hydrogel [73]. Noncovalent thermal gelation combined with covalent crosslinking with poly(caprolactone-co-lactide) (PCLA)-polyethylene glycol-PCLA triblock copolymers were used to incorporate celecoxib for its sustained release in joints [13].

Hydrogels with prolonged drug release in joints were created by combining noncovalent thermal gelation and covalent crosslinking with PCLA-PEG-PCLA triblock copolymers. The hybrid crosslinking technique allowed celecoxib to be loaded without sacrificing mechanical qualities. The hybrid crosslinked hydrogel released 20% celecoxib over 22 days, compared to 90% for a noncovalently crosslinked hydrogel [13].

Chitosan is also an excellent gelling agent and is biocompatible, biodegradable, nontoxic, and readily available. Several studies reported that chitosan was used as an in situ gelling agent. Chitosan could maintain the sustained release of dexamethasone as a thermosensitive gelling agent [74]. The chitosan-based thermosensitive hydrogel was used for the intra-articular administration of hyaluronic acid and corticosteroids. Hydrogels were composed of various ratios of chitosan and β-glycerophosphate. Polymer ratio and HA concentration affected gelation duration, swelling, and hydrogel surface shape [75]. A combination of chitosan and poloxamer was used to overcome the low bioavailability due to the short residence time of ciprofloxacin on ocular administration [76].

Gelation time and drug release from an in situ forming gel injectable can be efficiently modulated by varying the ratio of polymers used, storage condition, drug particle size, and solubilizers [77]. For clinically acceptable injectability, glucose-d-lactone and pyridoxal 5 phosphate ratios were altered in prefilled alginate solution combinations with paliperidone palmitate (PPP), CaCo3, and GDL. PPP was delivered in situ while creating a gel over four weeks in a regulated manner without an initial burst release [78].

## 5. Thermosensitive In Situ Gels Loaded with Micro and Nanoparticles

The IA administration of microparticles and nanoparticles is an approach that has been recently used for prolonging the residence of the administered drug within the joint cavity. These particulate systems include liposomes, microspheres, nanoparticles, and microcrystals. Since nanoparticles are quickly cleared from the joint cavity while microparticles are prone to phagocytosis by macrophages in synovial linings, an attempt was made to use in situ forming hydrogels as a vehicle to increase the retention time in joints [79]. Besides increasing drug retention at the site of injection, these nano or micro-sized particles also provide sustained release, thus prolonging the duration of action. In addition, such particles also help to minimize the initial burst effect, typically seen in the drug release profile of in situ gels.

Nanoparticles have also been used to improve the solubility of lipophilic drugs or increase the permeability through the synovial membrane. Below are a few instances where micro and nanocarriers have been investigated for their value addition to IA delivery of thermosensitive in situ gels. The composition and applications of some of these nanoparticles are given in Table 3.

A hyaluronan-based hydrogel formulation consisting of diclofenac-incorporated microspheres was found to increase the retention time of the drug. Lower amounts of the drug were needed to stabilize the therapeutic effect when compared to the conventional oral dose of the drug [80].

It was reported that exosomes have the potential to replace pharmaceuticals and stem cell-based treatments for osteoarthritis by decreasing cartilage degradation. A thermosensitive injectable hydrogel composed of Pluronic F127 and hyaluronic acid showed sustained release of exosomes and regulated chondrocyte proliferation, migration, and differentiation. M1 and M2 macrophage polarization was effectively induced [81].

Qi et al. prepared injectable thermosensitive hydrogels made of chitosan and β-glycerophosphate and dispersed them with diclofenac-sodium-loaded alginate microspheres. Drug release was sustained for up to 5 days, the swelling of the joints was reduced, and the symptoms of osteoarthritis were relieved [10].

Lornoxicam was intra-articularly administered as microspheres for osteoarthritis using chitosan/β-glycerophosphate-temperature-sensitive gelatine and PLGA. Microspheres exhibited sol-semisolid transition at 37 °C and turned to gel in 5 min, reducing the initial burst release. This approach delayed drug release with good drug retention and enhanced the drug targeting in the joint cavity [73].

Nanostructured lipid carriers (NLCs) were employed to encapsulate leflunomide, while PLGA nanoparticles were used as carriers for dexamethasone and loaded into chitosan β-glycerophosphate. The combination showed remarkable joint healing with a synergistic effect and prolonged release on IA administration [82].

A magnetic thermosensitive system composed of chitosan, β-glycerophosphate, Fe_3_O_4_, and mitomycin C as a chemotherapeutic agent was designed to instill the bladder via a catheter [94]. The formulation exhibited superior sol–gel transformation and magnetism. Mitomycin C was also released in a sustained manner and bladder retention was observed both in vitro and in vivo and lasted for 72 h in the rat.

Triamcinolone acetonide was loaded in thermoresponsive hydrogels with microparticles for the treatment of rheumatoid arthritis [83]. The in vitro release study confirmed the sustained release of the drug from hydrogels when compared to microparticles. In vivo studies proved the superiority of such integrated formulas in IA drug delivery. In another study, triamcinolone acetonide was encapsulated into temperature-dependent polymeric nanoparticles forming a sol–gel transition in an aqueous solution. On intra-articular administration, the formulation undergoes a rapid transition from a sol state to a viscous 3D hydrogel at body temperature with a sustained drug release for 6 weeks [85].

An in situ hydrogel composed of fibrin and hyaluronic acid was loaded with dexamethasone and galectin-3-inhibitor and formulated as nanocapsules. The system showed a sustained release of dexamethasone for 72 h in simulated synovial fluid. A combination of the in situ hydrogel with nanocapsule could be a suitable approach for arthropathy treatments [86].

Indomethacin and methotrexate were simultaneously delivered for the treatment of rheumatoid arthritis as temperature-sensitive hydrogel nanoparticles. Nanoparticles could effectively reduce joint swelling, bone erosion, and inflammatory cytokines in ankle and knee joint fluids with a sustained release of up to 72 h [87].

Dexamethasone was loaded in GE11-PLGA conjugate-based nanoparticles and then incorporated into a thermosetting chitosan-based hydrogel. After intra-articular administration, the dispersion was quickly converted from liquid to gel state within 15 min. The release of dexamethasone into the joint cavity was sustained [88].

An articular delivery system composed of poloxamer gel with clodronate in chitosan nanoparticles was developed for the controlled release of clodronate in joints. Nanoparticles loaded in poloxamer solution become gel at physiological temperatures with good anti-inflammatory activity [89].

Ethosomes were used as potential carriers to prolong the residence time of the drug in the knee joints. Ethosomes were inserted in a three-dimensional network of thermosensitive poloxamer gel (Etogel). The formulation was transformed into a gel at body temperature after injection into the joints [90].

A multifunctional thermosensitive hydrogel with poloxamer 407 and hyaluronic acid mixture as the gel matrix was incorporated with copper nanodots and platelet-rich plasma. On intra-articular administration, an in situ gel was formed which showed a slow release of copper nanodots, hyaluronic acid, and platelet-rich plasma [64].

Curcumin (Cu) was loaded in an NLC polymeric gel composed of cetyl palmitate, Labrafac PG, Captex 200, labrasol, and Tween 80 for intra-articular administration. Cu NLC gel was observed to undergo a sol–gel transformation at 33.21 °C with a significant reduction in knee joint inflammation in rats [91].

Hydrogels and nanoparticles were combined to develop a drug delivery platform of hydroxytyrosol, composed of hyaluronic acid and Pluronic F127. On injection, the thermosensitive formulation underwent a sol–gel transition at body temperature with resultant sustained action [92].

A chitosan-based thermosensitive hydrogel was developed and incorporated with Vancomycin nanoparticles to treat osteomyelitis. The vancomycin nanoparticle gel demonstrated prolonged drug release over 26 days, with enhanced osteoblast proliferation and rapid bone repair in rabbits [93].

## 6. Drug Release Mechanism from Thermoreversible In Situ Gels

After administration and phase transition, drug release from thermoreversible in situ gels cannot be different from other types of hydrogels. The possible release mechanism of drugs or therapeutics from thermoreversible gels is by passive diffusion with or without erosion or chemical reaction [95]. Various physical models have been studied to explain the diffusion of molecules through gels. It has been reported that entrapped molecules of different molecular sizes can be released by passive diffusion, and their movement depends on the mesh size of the swollen gel matrix [96].

Other factors that can influence the release behavior of the drug from the gel are the extent of crosslinking, presence of additives, structure and type of polymer, and conditions under which release takes place and the power of the external stimuli employed. Mason et al. reported a mesh size of 5–100 nm in swollen thermoreversible hydrogels, which is large enough to allow the diffusion of small drug molecules [97]. However, larger drug molecules will diffuse slowly in the swollen gel matrix, providing a sustained release. Gels from polymers with a greater degree of crosslinking tend to slow the diffusion of the drug through the matrix. Prince et al. prepared thermoresponsive gels by noncovalent thermal gelation and covalent crosslinking using poly(caprolactone-co-lactide) (PCLA)-poly (ethylene glycol) (PEG)-PCLA triblock copolymers.

**Figure 4:**
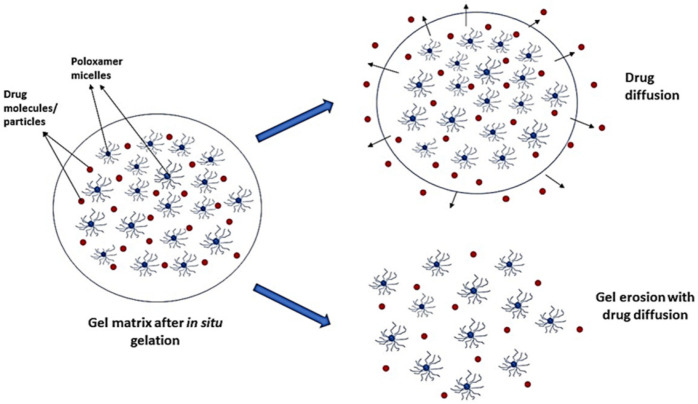
Drug release mechanisms from thermosensitive in situ gels.

Hybrid crosslinked hydrogels had higher viscoelastic properties and consequently slower drug release when compared to noncovalent crosslinked gels [13]. Poloxamer 407 and Poloxamer 188 are often used in combination in the formulation of thermoreversible gels. These two polymers are used in proportions necessary to provide a system with adequate gelling properties and the right phase transition temperature and adequate drug release [98]. This combination is also known to enhance the solubility of poorly soluble drugs and, consequently, their release [15]. Drug release from poloxamer gels is controlled by diffusion of the former through the gel matrix and erosion of the gel (Figure 4). The drug’s physicochemical properties and the gel’s composition determine which of these two processes will be the rate-limiting step in drug release [99,100].

Diffusion is favored when the entrapped drug molecules in the gel matrix are small. Besides the hydrophilic/lipophilic nature of the permeant, the extent of the water-filled channels also affects drug diffusion. It has been reported that drug diffusion tends to be slower with increasing poloxamer concentration due to a decrease in the size and number of aqueous channels arising out of an increase in the size and number of micelles [101]. Highly lipophilic drugs are released more slowly since they partition into the micelles in the gel structure [102].

Gel erosion is the rate-limiting step in drug release when the in situ gel has poorer mechanical strength, which causes degradation of the gel matrix and release of the incorporated drug. Hydrophobic or higher molecular weight drugs are also released by erosion of the gel matrix [100,103]. For instance, the release of acyclovir, a practical water-insoluble antiviral drug, from Poloxamer 407 gels was reported to be controlled by gel erosion [103]. The release became diffusion-controlled when carrageenan was incorporated into the poloxamer composition [103]. Hydrophobic drugs such as meloxicam, showed erosion-controlled release by zero-order kinetics from Poloxamer 188-based gels [104].

The inclusion of additives can alter the gel structure, gel porosity, and mechanical and rheological properties by hydrogen bonding and hydrophobic and supramolecular interactions. Researchers have used additives such as alginates [105], chitosan [106,107], carrageenan [103], gellan gum [108], cellulose derivatives [109], hyaluronic acid [110], and salts such as sodium chloride [104] into in situ gels to modify the release profile of the loaded drugs. These additives can change the gel erosion and diffusion characteristics of the thermoreversible gel.

Drug release from thermoreversible in situ gels can also be chemically controlled by the chemical reactions in the gel matrix. Hydrolysis or enzymatic degradation can bring about polymeric chain cleavage and reversible/irreversible reactions may also take place between the polymer network and the release of the drug [111].

As for most in situ gels, a major problem with thermoresponsive gels is a burst effect in drug release observed in the initial hours after injection into the body. This occurrence may be due to the slight delay between the injection and phase transition into the semisolid gel, which could produce systemic toxicity in the case of potent drugs [112].

## 7. Challenges in Formulation and Evaluation

Intra-articular in situ gelling formulations offer retention of drugs in the joints and allow for controlled release. Additionally, they share the advantages of the convenience of application, high drug loading, no organic solvents, sustained drug release behavior, and less systemic toxicity. While they claim the above benefits, the formulator must address some significant challenges when designing a high-quality in situ gelling drug delivery system [73].

Setting the gelation time as short as possible is equally important when optimizing the gelation temperature. Studies show that the gelation process can be sped up to 30 s. A longer gelation time may cause the drug to leach initially, interfering with the intended control release effect. The amount of gelation is indicated by an approximately 50-fold increase in viscosity from 8 to 37 °C. Poloxamer 407 and xanthan gum-based formulations had a viscosity of 2312 cps while in storage but increased to 69,198 cps viscosity when exposed to 37 °C (on gelling). In addition to having a low viscosity under storage conditions, the formulation should have sufficient syringeability. A formulation with a sheer thinning characteristic might be preferable for effective injection and syringe use. Achieving a linear drug release from the gels is highly desirable. Most of the in situ gel formulations follow a first-order release on in vitro evaluation. Biocompatibility, sterility, and adequate chemical stability of the formulation are also vital for any injectable formulation [5,72,73,82,113].

## 8. Stability of Thermosensitive Gels In Vitro and In Vivo with Biodegradation

It has been reported that nonbiodegradable implant materials used in bone grafting may infect the site. Therefore, the ideal biomaterials for clinical use provide adequate biocompatibility and an acceptable degradation profile [114]. The stability and biodegradability of implant hydrogels are vital since these two parameters control the drug release rate and treatment efficacy. Gels get their three-dimensional structure and mechanical strength from the crosslinking agents [115]. Until the drug is fully released to the site, the hydrogel ought to remain stable. The three-dimensional structure extends the drug’s half-life, enhances therapeutic efficacy, and inhibits early drug degradation by body fluid [116]. An in vivo investigation (Hu Z et al.) showed that the implanted hydrogel took ten weeks to erode the structure. The gel structure is intact at the beginning of the therapy and gradually dissolves after six weeks. This results in the more efficient delivery of the drug [117].

One of the critical factors that influence the stability of thermosensitive gels is viscosity. Polymers that are both hydrophilic and hydrophobic make up the gel structure. High-viscosity hydrogels displayed a slower loss of gel mass. Because of decreased body fluid penetration and hydration, hydrophobic hydrogels break down more slowly [118].

Ruan H. and colleagues revealed in 2018 that at the point of application of the drug, a tiny quantity (5%) of the drug is degraded and lost by uncontrolled release from the gel structure. The drug release of in situ gels was gradual, with initial burst effects continued by sustained release. The initial release is due to the formulation’s sol–gel transition [119]. The gelation properties of polymers were impacted as a result of their degradation. The viscosity of the gel drastically decreases as a result of the polymer breaking down in the system. The shift in viscosity can result in an increased rate of medication delivery to the affected area. Since this is the case, it is usually preferable that the polymer suitable for the formulation of the implant matrix solution has high gelling strength and a moderate degradation rate [120]. The timing of polymer resorption should coincide with the rate of bone tissue regrowth. This helps to efficiently administer the medicine throughout the course of treatment and provides support for the bone material to grow as fillers on the surface pores of the disintegrating gel [121].

## 9. Clinical Applications of Intra-Articular Hydrogels

Osteoarthritis and associated inflammation are treated by intra-articular HA administration. The frequency of administration varies with the rate of degradation of HA released form the formulation into the joint [30]. Several manufacturers are marketing in situ gelling injections claiming extended release for the HA for a longer duration of action. Gel-One^®^ [122], a PVA-based hydrogel named Synvisc-One^®^ [123], and KD Intra-articular Gel^®^ are examples. The gelation mechanisms involved in these gels are not discussed in the public domain.

## 10. Conclusions

Joint diseases such as RA and OA can be quite debilitating and cause severe distress to the sufferer. Although several therapeutics are available to control these diseases, such as NSAIDs and corticosteroids, the IA administration of the traditional form of these drugs is inefficient in the long-term management of chronic inflammatory disorders of the joints. Recent FDA approvals of various intra-articular gels such as Durolane—Bioventus^®^ (hyaluronic acid gel) and Zilretta—Flexion^®^ (triamcinolone acetonide) clearly demonstrate the market need for sustained-release formulations for intra-articular therapy. In stimuli-responsive gelling systems, the drug effects can be sustained due to the depot effect of the formulation after phase transition. Thermoreversible gelling solutions can be considered valuable vehicles in the IA administration of therapeutics with the advantages of accuracy and ease of administration, as with any injectable solution. The gelation at body temperature after IA injection can minimize rapid clearance of the drug from the joint cavity and prolong its release. The most critical factors determining the release rate of drugs from the gel matrix are the molecular size of the permeant, composition, concentration, and properties of the thermosensitive polymer base. Drug release may take place by passive diffusion or erosion or both. Thermosensitive hydrogels are more stable and unaffected by pH than other stimuli-sensitive in situ gels. Temperature-based gelling systems are also the most reliable as their properties do not vary much between individuals, whereas there is a lack of predictability with other stimuli which do not remain constant in pathological conditions. The challenges associated with thermoreversible gels such as optimization of gelation temperature by changing polymer composition, biocompatibility, sterilization difficulties, and effect of storage conditions on the shear-thinning properties must be considered when formulating an optimal system.

## Figures and Tables

**Table 1:** Types of intra-articular treatments used in the management of joint disorders.

	NSAIDs	Hyaluronic Acid	Corticosteroids	Platelet-Rich Plasma	Mesenchymal Stem Cells
Constituents	Aspirin, Ibuprofen, Naproxen, Celecoxib, Diclofenac, Ketoprofen	Hyaluronic acid	Betamethasone acetate, Dexamethasone acetate, Triamcinolone hexacetonide, Betamethasone sodium phosphate, Prednisone tebutate	Cells and coagulation factors, anticoagulants, fibrinogen, activators, platelet-rich fibrin, leukocyte-rich plasma	Suspension of mesenchymal stem cells
Advantages	Inexpensive Noninfectious Used as monotherapy	Relatively safe Benefit up to 60 days	Low dose is required: eg. triamcinolone acetonide (5 mg). Adverse effect is very low Provide pain relief and reduce joint effusions	Simple Low cost Minimally invasive Reduce inflammation, pain relief, improved function, and possible cartilage regeneration	Safe and encouraging results for articular cartilage repair and regeneration
Disadvantages	Dose-dependent toxicity. Using NSAIDs in the short term. Long-term usage may cause liver toxicity	Risk of infection Local adverse events after injection due to injection technique, partially flexed knee, etc. Other side effects are acute synovitis, joint swelling for up to 3 weeks, haemarthrosis, pseudogout, muscle pain	Short-lived beneficial effect: not more than one week. IA corticosteroids seem to produce time and dose-dependent deleterious effects on articular cartilage with erosion, decreased glycosaminoglycans content, and joint narrowing Local side effects include post-injection flare, infection, and skin hypopigmentation	PRP formulations are complex Mechanisms of action in a joint with OA remain unanswered. More clinical evidence required Optimal therapeutic protocol not yet been established related to timing, dosage, volume, frequency, and composition	Preparation is complex Exact mechanism of action of MSCs is debated Long-term clinical trial studies are required
References	[21,22,26,27]	[26,31,32,33]	[19,23,46,47]	[26,39,41,47]	[26,37,40,42]

**Table 2:** Stimuli-responsive hydrogels with their advantages and limitations.

Type of Stimuli-Responsive Hydrogel	Advantages	Limitations	References
Thermosensitive	Compatible with both hydrophilic and hydrophobic drugs, high drug loading capacity, drug sustained-release carriers, easy to formulate with novel drug delivery carrier systems	Long responsive time and low biocompatibility	[51]
Magnetic-responsive	Biocompatibility, controlled architectures, smart response to the magnetic field	Long response time and less precision in drug release	[59]
Ultrasound-activated	Suitable with various drug delivery carriers	Damage to the surrounding tissue	[60]
Enzyme-responsive	Offers specificity towards degrading enzymes. Quick degradation and drug release in presence of specific enzymes	Quick degradation limits the usefulness of long-term therapy	[61]
pH-responsive	Altered tissue pH in pathological conditions is effectively utilized for gelation at selective sites of tissue inflammation such as cancer	pH variations in pathological conditions may adversely affect the gel	[61]

**Table 3:** Nanostructures used in thermosensitive gels and their applications.

Nanosystems	Composition	Applications	References
Collagomers	Collagen type I, dipalmitoyl phosphatidylethanolamine, glutaraldehyde	Osteoarthritis	[80]
Hydrogel + exosomes	Pluronic F 127, hyaluronic acid	Osteoarthritis	[81]
Microspheres	Poloxamer 407, alginate sodium, chitosan, β glycerophosphate	Rheumatoid arthritis	[10]
Hydrogel	Chitosan, β glycerophosphate	Rheumatoid arthritis	[82]
Microparticles	Polyethylene glycol methacrylate methyl ether (PEGMA 246, 188, 475)	Rheumatoid arthritis	[83]
Hydrogel	PEG1500_,_ ε-caprolactone, tri ethyl amine, toluene	Osteoarthritis	[84]
Polymeric-nanoparticle-based hydrogel system	Tetrahydrofuran, trimethyl- amine, Methoxy poly (ethylene glycol)	Osteoarthritis	[85]
Hydrogel loaded in nanocapsules	Soya lecithin, oleylamine, olive oil, ethanol,	Joint diseases	[86]
Hydrogel nanoparticles	Polyethyleneimine, Pluronic F 127 and 68, propylene sulfide	Rheumatoid arthritis	[87]
PLGA-based nanoparticles	GE11, PLGA, PEG, chitosan, β-glycerophosphate	Joint diseases	[88]
Polymeric nanoparticles	Chitosan, clodronate, TPP, glutaraldehyde	Rheumatoid arthritis	[89]
Ethosomes	Phospholipon 90 G, ethanol	Joint diseases	[90]
Hydrogel	Poloxamer 407, hyaluronic acid	Osteoarthritis	[64]
NLC-based gels	Cetyl palmitate, Labrafac PG, Captex 200, Tween 80, Labrasol, Pluronic F 68 and 127	Rheumatoid arthritis	[91]
In situ hydrogel + nanoparticles	Hyaluronic acid, chitosan, Pluronic F 127	Osteoarthritis	[92]
Hydrogel	Chitosan, quaternaryammonium chitosan, β-glycerol phosphate disodium	Osteomylitis	[93]
